# Decontamination of Mycotoxin-Contaminated Feedstuffs and Compound Feed

**DOI:** 10.3390/toxins11110617

**Published:** 2019-10-25

**Authors:** Radmilo Čolović, Nikola Puvača, Federica Cheli, Giuseppina Avantaggiato, Donato Greco, Olivera Đuragić, Jovana Kos, Luciano Pinotti

**Affiliations:** 1 Institute of Food Technology, University of Novi Sad, Bulevar cara Lazara, 21000 Novi Sad, Serbia; olivera.djuragic@fins.uns.ac.rs (O.Đ.); jovana.kos@fins.uns.ac.rs (J.K.); 2 Department of Engineering Management in Biotechnology, Faculty of Economics and Engineering Management in Novi Sad, University Business Academy in Novi Sad, Cvećarska, 21000 Novi Sad, Serbia; 3 Department of Health, Animal Science and Food Safety, University of Milan, Via Trentacoste, 20134 Milan, Italy; luciano.pinotti@unimi.it; 4 Institute of Sciences of Food Production (ISPA), National Research Council (CNR), Via Amendola, 70126 Bari, Italy; giuseppina.avantaggiato@ispa.cnr.it (G.A.); greco_donato@libero.it (D.G.)

**Keywords:** mycotoxins, reduction, grain cleaning, thermal processing, chemicals, adsorbents

## Abstract

Mycotoxins are known worldwide as fungus-produced toxins that adulterate a wide heterogeneity of raw feed ingredients and final products. Consumption of mycotoxins-contaminated feed causes a plethora of harmful responses from acute toxicity to many persistent health disorders with lethal outcomes; such as mycotoxicosis when ingested by animals. Therefore, the main task for feed producers is to minimize the concentration of mycotoxin by applying different strategies aimed at minimizing the risk of mycotoxin effects on animals and human health. Once mycotoxins enter the production chain it is hard to eliminate or inactivate them. This paper examines the most recent findings on different processes and strategies for the reduction of toxicity of mycotoxins in animals. The review gives detailed information about the decontamination approaches to mitigate mycotoxin contamination of feedstuffs and compound feed, which could be implemented in practice.

## 1. Introduction

Modern feed mills produce a wide range of products on a daily basis, regardless of whether they have one or several processing lines. Formulated diets are often composed of more than 20 ingredients and each of the ingredients is carefully selected based on the nutritional quality, safety, price, and availability [1]. Safe ingredients are important for the production of safe animal feed, which is in turn important for animal health, production of safe animal products for human consumption, and for the environment. To ensure security in the agro-food chain, the feed mills are obliged to control all raw materials and products for the presence of possible contaminants as well as to test numerous samples on a daily basis [2]. Mycotoxins are a major contaminant of feed ingredients and products. Since these secondary metabolites of molds are toxic, feed producers have to ensure that concentrations of these contaminants do not exceed maximum allowed values for a specific mycotoxin. The occurrence of mycotoxin is a significant global challenge, accompanied by rising animal and human health hazards and huge financial losses in the food and feed production industries [3,4].

Different methods are used to decontaminate mycotoxin-contaminated commodities or to reduce the exposure to mycotoxins, but not all approaches are appropriate for feed and compound feed manufacturers. An efficient method for the reduction of mycotoxins should be able to remove or inactivate the mycotoxins without producing toxic residues and affecting the technological properties, nutritive value, and palatability of products [5]. Modern feed production is mainly done on a large scale, and proposed strategies should also be capable of being implemented on a large scale as well [6]. Due to the high cost of raw materials, which contribute up to 70% of the costs of compound feed, feed production costs are optimized, and only relatively simple and/or inexpensive strategies are acceptable for mycotoxin removal [7].

Essential oils are biological technologies for the decontamination of mycotoxins in feedstuffs. The oils present secondary metabolites extracted from plants and consist primarily of monoterpenes and sesquiterpenes and phenylpropanoids, compounds responsible for the oil’s organoleptic characteristics [8]. Fungi metabolize and synthesize many organic compounds with bad properties during their life cycle [9].

Many physical, chemical and biological technologies have been suggested with the aim of reducing unavoidable and unpredictable mycotoxin contamination and many have shown excellent effectiveness. This article reviews the latest results on various procedures and strategies used to reduce mycotoxin contamination in the feedstuffs and compound feed, as well as to reduce the toxicity of mycotoxins in animals.

## 2. Grain Cleaning

Grains are normally received in bulk from receiving hoppers of feed mills. Since the unloading area is the largest source for dust emissions, and since there is an explosion risk, this is the place where the dust control equipment is installed. Also, mills are often equipped with vibratory screens, or other grain cleaning equipment, which removes oversized or undersized material [10]. These processing steps are introduced to remove foreign material, such as stalks, paper, wood scraps, etc., but they have also shown to be effective in mycotoxin removal. The mycotoxin concentration can be decreased by removing kernels with mold growth, crushed kernels and dust [11].

Cereals, which are a major part of animal diets, are often received in a feed mill in grain form. Depending on the climate conditions in the location where the crops are grown, cereals might be a favorable substrate for mycotoxigenic fungal species. These ingredients have high inclusion rates in animal compound feed, and if contaminated, could be a source of contamination of the final products [12,13]. Therefore, the focus of numerous studies was the investigation of procedures for decontamination of cereal grains [14,15,16,17,18,19,20].

Not all parts of the grain kernels are susceptible to fungal contamination. The outer parts of grains, such as germ and pericarp, have a higher tendency to be contaminated by mycotoxins than the endosperm [21,22]. The data from the literature confirms that the milling process redirects the mycotoxins into inedible byproducts, but these byproducts are commonly used as animal feed, such as bran, feed flour, polishings, etc. [23,24]. Also, dehulling, scouring or other processes where a part of the grain is removed are not typical for the animal feed production process. When the whole grains are used in the feed mill, the most commonly used grinding equipment is a hammer mill, roller mill, pulverizer, disk mill, etc. All of these machines grind a whole cereal grain without subsequent fractionation and possible redirection of mycotoxins [25].

Dust removal can be efficient in removing mycotoxins. As shown by different studies, mycotoxins are often accumulated in the grain dust, and exposure to it can have toxic effects on animals and humans [26,27]. Efficient dust collection systems and separation systems can be efficient in mycotoxin redirection into fine fractions which are excluded from the raw materials or final products. Different dust separation systems might be implemented in the feed mill. Some of the installed options have the possibility to adjust the cutting point for the separation of fine particles in wider ranges of particle sizes. Vidosavljević et al. [28] have applied gravitational cascade zig-zag classifier for dust removal, and have been able to reach the level of aflatoxin reduction higher than 90% in the coarse fraction. Higher airspeed might be more efficient in mycotoxin removal, but they also increase the yield of the fine fraction which is considered a waste product [28]. In addition, separation systems can also be used for the removal of broken kernels. Broken kernels might be a potential source of mycotoxin contamination since they have a higher mycotoxin level than whole kernels. Screening and gravity are used more frequently than air separation/classification for the separation of broken kernels and coarse impurities separation. Dry cleaning of grain surface may also lead to a reduction of molds and mycotoxins. For example, it has been demonstrated that deoxynivalenol content can be reduced up to 84%, while the aflatoxin level was reduced by approximately 62% by the use of polypropylene bristle brushes for polishing of grain surface without damage of pericarp [28]. The increase in the retention time of contaminated material in the cleaning device and cleaning intensity increases the effectiveness of mycotoxin removal [28,29]. However, these processing steps are not commonly used in the processing line in order to avoid potential bottlenecks.

In cases where the size of contaminated kernels is similar to the size of whole kernels, typical separation technologies based on a difference in a mass or density are not very efficient in the removal of infected material [29]. Since these infected grains can be visually differentiated from the healthy grains, their separation from the intact commodity can be effective in mycotoxin reduction. It has been reported that even manual sorting can be effective in aflatoxin, deoxynivalenol, and fumonisin reduction by up to 80%, 83.6%, and 84%, respectively [28,30]. Taking into account that the average feed mill capacity is approx. 50,000 tons per year, it is clear why it is not possible to implement manual sorting at a large scale. On the other hand, optical sorting has been successfully implemented at a large scale in the feed industry for the removal of infected kernels based on color difference. Within the optical sorting machines, grain streams are directed along by optical sensors and the grains different in color were removed by a jet of pressurized air from the stream. By removing infected grains, it is also possible to reduce mycotoxin content by more than 80% [14,31].

Other physical treatments that have shown to be effective in mycotoxin decontamination are flotation in various solutions, cold plasma, gamma-radiation, microwave heating, etc. They are not commonly used by the feed industry due to different reasons, such as high investment cost, high operational costs, unavailable processing units on a large scale required by the feed producers, etc.

## 3. Thermal Processing

In general, mycotoxins are mainly stable compounds under conditions of thermal processes that are most commonly used in food and feed production [11,32]. The following factors of thermal processing are the most important for the degradation and reduction of mycotoxins in food and feed: type of mycotoxin, the initial mycotoxin concentration, temperature, time of exposure to high temperature, the degree of heat penetration, pH, moisture content, etc. [32]. If raw materials are contaminated with some regulated and/or non-regulated mycotoxins there is a great possibility that final products will also contain those mycotoxins, since they are not completely destroyed during the applied thermal process. Different processes of thermal food and feed treatment that can have different impacts on mycotoxins include extrusion, cooking, frying, baking, canning, crumbling, pelleting, roasting, flaking, nixtamalization, alkaline cooking, etc. [11]. Only a few of the listed processes, such as crumbling, pelleting and extrusion are commonly used in compound feed preparation as well as in feed production. Even though these processes can significantly reduce the mycotoxin concentration, their implementation usually does not lead to the complete elimination of mycotoxins. Among the thermal treatments, the utilization of high-temperature processes demonstrated the greatest potential for mycotoxins reduction [11]. Kabak [32] examined the effect of different extrusion parameters on the reduction of some regulated mycotoxins. He concluded that the application of extrusion at a temperature higher than 150 °C have a significant impact on the reduction of zearalenone and fumonisins, while the same conditions led to moderate reduction of aflatoxins and deoxynivalenol. The extrusion process with temperatures at around 160 °C or higher, in combination with glucose, demonstrated the greatest degree of reduction of fumonisins. For example, after the extrusion of corn grits with 10% added glucose, initial fumonisin B_1_ concentration was reduced by 75–85%. In addition to fumonisin reduction, the applied extrusion process resulted in the formation of high amounts of N-(1-deoxy-d-fructos-1-yl)-Fumonisin B_1_, and in small amounts of hydrolyzed fumonisin B_1_ and N-(Carboxymethyl)—Fumonisin B_1_. Fumonisins contaminated corn grits extruded with glucose demonstrated lower toxicity during feeding trial toxicity tests in rats [33]. However, it is important to note that the process for the production of animal feed includes mixing different batches of different raw ingredients. Therefore, creating a new complex feed mixture represents a great risk since each raw ingredient has its own initial contamination with an entirely new risk profile for the majority of mycotoxins [34]. Roasting and extrusion processing, reaching temperatures of 150 °C or more, could contribute to the reduction of contaminated batches [15,18,28]. In this complex scenario, further investigation related to the fate of various mycotoxins during thermal feed processing, as well as their overall loss in toxicity in different feed matrixes is clearly required in order to define the best thermal processes for mycotoxins reductions on one hand, and to avoid losses of feed nutritional quality on the other hand. However, the application of high temperatures in feed processes can have variable effects on mycotoxins, from significant to slight reduction.

## 4. Chemical Agents

In spite of all positive sides of available chemical treatments for decontamination of mycotoxin contaminated feedstuffs and compound feed, their limitations are also present, since the products handled must be safe from the chemicals used and the nutritional value of the products should not be altered or deteriorated [15]. Not all agents are efficient to the same degree against mycotoxins, but science is still making efforts to find a broad variety of chemicals that will be effective on a higher scale against a larger number of mycotoxins [16]. Nowadays, there are several chemicals agents used for mycotoxin contaminations mycotoxin decontaminations and can be divided into categories such as alkaline (Ammonia gas NH_4_OH; Sodium hydroxide NaOH; Calcium hydroxide Ca(OH)_2_), acids (Acetic acid C_2_H_3_OH; Phosphoric acid H_3_PO_4_; Formic acid CH_2_O_2_; Propionic acid CH_3_CH_2_COOH; Sorbic acid C_6_H_8_O_2_; Sodium hypochlorite NaClO), reducing agents (Sodium bisulfite NaHSO_3_; Sugars: D-glucose or D-fructose), oxidising reagents (Ozone O_3_; Hydrogen peroxide H_2_O_2_), and many others such as chlorinating agents, salts and miscellaneous reagents [15,17].

Cereals treatment with ammonia gas is known as the method of ammoniation, which gained great attention in detoxification of aflatoxin and ochratoxin, and have been used for decontamination in several countries [16,17]. However, the efficacy of ammoniation varies on the type of mycotoxin. The efficiency of applied NH_4_OH on laboratory animals with the purpose of lowering concentrations of fumonisins did not show any promising results since there was no decrease in toxicity when ammonia fumonisin B_1_ was supplied to livestock despite a decrease in fumonisin B_1_ concentration. In the last several years in developed countries, ammoniation has been successfully used in maize grain decontaminations, in particular, to reduce the amount of contamination of aflatoxins in feed [14,18]. Ammoniation is usually the most effective against aflatoxin B_1_, with the remaining side product of aflatoxin D_1_, which is far less toxic than the aflatoxin B_1_. In addition, the positive effects of ammoniation in feedstuffs and compound feed detoxification could be compared to the high cost of applied methodology, and more the ineffectiveness of the method against other mycotoxins. Nevertheless, this method can lead to food quality decrease and deterioration due to involved excessive ammonia levels in the food [35]. Calcium hydroxide was used to decontaminate feeds contaminated with T-2 toxin and diacetoxyscirpenol. Under the aforementioned alkaline conditions, mycotoxin structure can certainly be changed [19].

Feedstuffs and compound feed treatment with strong acids could destroy the biological activity of aflatoxin B_1_ Thus converting aflatoxin B1 to a compound which is hemiacetal or a hemiketal compound the results from the addition of an alcohol to an aldehyde or a ketone, which are formed when a second alkoxy group has been added to the structure, respectively [36]. HCl treatment (pH 2) showed a 19.3% reduction in aflatoxin B_1_ concentrations within 24 h. Formic, propionic and sorbic acids show their positive influence when it comes to the degradation of ochratoxin A with concentrations ranging from 0.25 to 1.0% after exposure to this particular acid during the time which is no longer of 24 h. Sodium hypochlorite can be used successfully in the destruction of ochratoxin A as a pale greenish-yellow dilute solution commonly known as liquid bleach or simply bleach [37].

Reducing agents such as sodium bisulfite have the affinity to react with aflatoxins and trichothecenes. Their mechanism of action includes the formation of sulphonate derivatives while peroxide and heat enhance the destruction of aflatoxin B_1_ by sodium bisulfite [38]. Besides aflatoxin B_1_ and trichothecenes, reducing agents decreased the levels of deoxynivalenol as well. The conversion of sodium bisulfite from deoxynivalenol to deoxynivalenol-sulfonate, which is less toxic than deoxynivalenol, has been recorded as an efficient instrument to overcome the depressive impacts of deoxynivalenol on feed consumption in certain species and farm animal categories. Temperatures at approximately 65 °C for 48 h can block the primary amino group of fumonisin B_1_ and prevent toxicity of cell tissue cultures of farm and laboratory animals caused by the presence of fumonisin in feed, but only in the presence of D-glucose or D-fructose sugar reduction [39].

Oxidizing agents such as ozone and hydrogen peroxide were used to decontaminate mycotoxin contaminated raw feed and compound feed [40]. In addition to feed and compound feed, ozone treatment has been used with very high success over the years to decontaminate food products. Many of the chemical methods mentioned above could be used to reduce mycotoxin levels in feed and compound foods with a high percentage of efficacy, but it could not be neglected that these chemicals could potentially activate changes in the nutritional, physical and sensory properties of treated materials [41]. Protection against aflatoxin B_1_ in poultry has been proven in the research where it has been shown that chemically oxidizing agents react with a wide range of different functional groups, where aflatoxin B1 contaminated corn was treated with electrochemically produced ozone. Other research has also proved the positive effects of the ozone when a contaminated cereal with ochratoxin A was treated [42]. Ozone possesses the ability to reduce mycotoxin contamination and improve microbiological status. When 10% of hydrogen peroxide (H_2_O_2_) was used to decontaminate zearalenone contaminated grains during a period of 16 h at a temperature of 80 °C, a degradation of 84% was recorded. The elevated degree of contamination of feedstuffs by these microorganisms has resulted in significant losses to enterprises as these microorganisms generate mycotoxins on a massive scale, in addition to the decay of the raw materials. *Aspergillus carbonarius* and *Aspergillus niger* produce ochratoxin A trough their secondary metabolism [43].

The use of both physical or chemical processes outlined earlier to decontaminate feed and compound feed is restricted by very high expenses and some nutrient quality losses. Scientists have reached the concept of detoxifying mycotoxins through biological conversion, which can be described as the degradation or enzymatic conversion of mycotoxins into less toxic compounds.

## 5. Feed Additives for the Prevention of Mycotoxin Effects

Feed additives are mixed with contaminated diet to minimize the effect of mycotoxins on the animal prior to intake or during digestion [44,45].

The use of feed additives or supplements that decrease animal exposure to mycotoxins can be viewed as a means of enhancing animal welfare. These feed supplements are referred to as the substances blended into feed (e.g., mineral clay, micro-organism, yeast cell wall), adsorbing or detoxifying mycotoxins in the digestive tract of animals (biological detoxification) [46]. These additives have received increasing attention from the feed industry and numerous products have been developed and some of them have already been tested on animals and marketed.

European Regulation (EC) No 1831/2003 of 22 September 2003 on animal feed additives has been revised and the category of technological feed additives includes a special functional group [47]. That is a group which is described as “substances that can suppress or decrease the absorption of food through mycotoxins, encourage the excretion of mycotoxins or alter their mode of action”, under Commission Regulation (EC) No 386/2009 of 12 May 2009 [48]. It should be pointed out that the use of such products does not mean that the animal feed exceeding the established maximum limits may be used. Their use should rather improve the quality of the feed which is lawfully on the market, providing an additional guarantee for the protection of animal and public health. Therefore, after adding an additive, these additives may not be used as compatible in non-conforming camouflage consignments. Following a request for technical assistance, in July 2010, the European Food Safety Authority (EFSA) through its Panel on Additives and Products or Substances used in Animal Feed (FEEDAP) issued a statement where it detailed the additional information that would be required to perform an assessment of safety and efficacy of this new group of additives [49]. This statement lists only the requirements which are not common in relation to the rest of technological additives. In 2012, EFSA published several guidelines on its website pertaining to the marketing of several feed additives and safety measures [50]. This guidance document follows the structure and definitions of Regulation (EC) No 1831/2003 and it is intended to assist the applicant in the preparation and the presentation of its application, as foreseen in Article 7.6 of Regulation (EC) No 1831/2003 [48].

Various materials have been tested as mycotoxin-detoxifying agents in order to avoid deleterious impacts of mycotoxins on livestock (mainly poultry and swine). They work either in adsorption or in the bonding or transformation of mycotoxins to their surfaces (biotransformation), depending on their mode of action. Biotransformation of mycotoxins can be caused by the addition of enzymes or micro-organisms generating such enzymes [46].

Mycotoxin binders are nutritionally inert adsorbents that reduce mycotoxin absorption from the gastrointestinal tract by integrating them into contaminated feed, thereby preventing and decreasing mycotoxicosis and transportation of mycotoxins into animal products [46]. The adsorbent materials are designed to behave like a “chemical sponge”, preventing the blood and target organ absorption and later distribution of mycotoxins. The effectiveness of adsorbent on mycotoxin seems to depend on the chemical structure. The main feature is the physical adsorbent structure, i.e., the total distribution of load and load, the dimensions of pores and the available surface. On the other side, adsorbing mycotoxins also have a major part to play, such as polarity, solubility, form and load distribution. The stability of the sorbent toxin bond and the efficacy over a broad pH range are important criteria for the assessment of possible mycotoxin binders because a product has to be implemented on the entire gastrointestinal tract [51]. The feed composition can also have a major impact on adsorption effectiveness [51]. Potential absorbent materials include activated carbon, aluminosilicates (bentonite, zeolite, phyllosilicates, etc.), complex indigestible carbohydrates (cellulose, polysaccharides in the cell walls of yeast and bacteria such as glucomannans, peptidoglycans, and others), and synthetic polymers such as cholestyramine and polyvinylpyrrolidone and derivatives [20,45,46,52,53,54]. Many studies have shown that the formation of stable connections of these adsorbent products has a strong affinity with mycotoxins. These are found in a number of fluid systems, such as beer, wine, milk, and peanut oil.

Activated carbon is a widely used adsorption material that has an outstanding adsorption ability with a wide surface region. It is recommended for multiple digestive toxins as a general toxic adsorbing agent and is frequently suggested (The Merck Veterinary Manual, Eighth Edition, Merck & Co., Inc., Whitehouse Station, NJ) [55]. Activated carbon effectiveness depends on the source materials, the surface area and the distribution of the pores on the adsorption characteristics of the activated carbon. The surface features of activated carbons are greatly altered by preparation techniques and chemical treatments. The contrasting findings regarding the capacity of activated carbon for mycotoxin binding can explain different adsorbing characteristics of different carbonaceous materials [51]. Activated carbon adsorbs most mycotoxins effectively in water, whereas animals are less or not affected by mycotoxicosis. For aflatoxin B_1_ and ochratoxin A adsorption, the highest capacity for in vitro activated carbon was noted whereas deoxynivalenol adsorption was lower. The efficacy of activated carbon has been demonstrated in vivo and in vitro by vibrant gastrointestinal models for deoxynivalenol, nivalenol, zearalenone, aflatoxin, ochratoxin A, diacetoxyscirpenol and T-2 toxins [44,53,56,57,58,59,60]. Responses to charcoal in cows, broilers, turkey poults, rats and mink suggest that charcoal may not be as effective in binding aflatoxin as the clay-based binders. The biomarker assay in rats did not confirm the in vivo efficacy of activated carbon to bind fumonisin [52,61]. Also, research conducted with weaning pigs showed that they were not effectively protected against the adverse effects of consuming fumonisin B_1_ by adding activated carbon to contaminated feed [62]. Finally, although having a potential for acute exposure to a number of mycotoxins, activated carbon is a non-specific sequester with large variability in efficacy, which reduces possibilities for its practical application.

Mycotoxin binders are the largest and most complicated class of silicate minerals [63,64,65,66,67]. There are two major sub-classes in this group, phyllosilicate, and tectosilicate [68,69]. The phyllosilicate sub-class mineral clays include significant adsorbents such as the montmorillonite/smectite group, the kaolinite group and the illite (or clay-mica) group [51]. Montmorillonite is a predominantly layered, oxygen-coordinated, phyllosilicate consisting of octahedral aluminum and tetrahedral silicon layers. The bentonite is usually impure smectite clay. The tectosilicates include important and highly studied zeolites. Zeolites consist of SiO_4_ and AlO_4_ tetrahedrons having a cage-like structure that is infinite in three dimensions. In such minerals, some tetravalent silicones are replaced by trivalent aluminum, which results in inorganic cations, such as sodium, calcium and potassium ions, that have a lack of a positive charge. Clay minerals, primarily montmorillonite, have been used in the early 1970s to reduce aflatoxin toxicity [70]. There is ample literature on this subject, mainly in the field of in vitro water studies [53,63,64,66,71], and animal feed trials [20,72,73,74]. The use of smectite in human nutrition was also tested for its safety as well as the efficacy in the decrease of aflatoxin biomarkers [64,75,76,77]. In Europe, bentonite is allowed as a feed additive for all animal species, as well as for mitigation of mycotoxin contamination for ruminants, swine, and poultry (1m558). It is also used for control of radionuclide contamination and as an anticaking agent (1m558*i*). The chemisorption of aflatoxin to smectites involves the formation of a complex by the β-keto-lactone or bilactone system of aflatoxin with uncoordinated metal ions in the mineral. Aflatoxin B_1_ is able to be attached on the surface of the mineral particle and in its interlayers. A huge difference in the effectiveness of bentonites in sequestration of aflatoxin B_1_ was shown in several in vitro studies [51]. These studies indicated that aflatoxin B_1_ bentonite adsorption efficacy may rely on the physical, chemical and mineralogical characteristics of the smectite, including clay contents, the capacities of the cation exchange (CECs), the interlayer cation hydrate radius, distributions of particle size and the specific surface area. Notwithstanding these findings, an important correlation has not been well created between smectites minero-chemical and physicochemical characteristics and aflatoxin B_1_ adsorption. Therefore, there is still no predictive model of aflatoxin B_1_ adsorption by the bentonite as the crystal-chemical variation in the smectite group is complex. Recently, the study by D’Ascanio et al. [67] showed a strong correlation between aflatoxin adsorption parameters and the geological origin of samples. In adsorbing toxin at distinct pH values, sedimentary bentonites were considerably better than hydrothermal bentonites [51]. The extent of aflatoxin B_1_-adsorption was negative and linear with the extent of desorption. Mineralogical and physicochemical analyses confirmed that some physical and chemical properties of bentonites correlate linearly with AFB_1_ adsorption. However, these studies cannot be deemed to be conclusive since it is still hard to depict the link between properties of these mineral adsorbents and aflatoxin B_1_ adsorption/desorption. Due to the complexity of interactions and factors that can affect the adsorption of the aflatoxins by smectites, further research is required to describe the mechanisms of adsorption [51].

However, bentonite cannot be used as a binder for all mycotoxins due to their limited binding effects. Several in vivo studies have previously shown that aluminosilicates do not significantly adsorb other mycotoxins, such as cyclopiazonic acid and ergotamine, zearalenone, deoxynivalenol, T-2 toxin, ochratoxin A and others. The selective chemisorption of bentonites for aflatoxins can be overcome by chemical modifications. These include changes in the surface characteristics, resulting in enhanced hydrophobicity when structural load balancing cations are exchanged with molecular heavyweight amines [66,78]. Several in vitro studies showed the binding efficacy of modified montmorillonite and clinoptilolite against zearalenone and ochratoxin A [20]. Aflatoxin B_1_ was adsorbed with non-modified zeolites. However, the in vivo ineffectiveness of these binders in sequestering a large spectrum of mycotoxins has been recently observed in piglets [79], and some of those clay forms were pointed out to the potential toxicity [80,81].

Recently, questions have been raised about the nanotechnology solution of mycotoxin risk [82]. One of the most promising methods is the use of carbon-based nanomaterials. Graphene has shown a huge surface and a high mycotoxin binding capacity. Polymeric nanoparticles have also been drawn to attention; they may replace adsorbents or contain a substance that would improve the organism’s health status. Modified nanodiamonds synthesized by detonation were proposed as intestinal adsorbent of aflatoxins [83]. Highly advanced surfaces, and the existence on the surface of nanoparticles of multiple functional chemical-active groups, hydrocarbon fragments, and metal micro impurities, establish their elevated affinity to biomolecular sorption. The findings of in vitro experiments, showing that nanodiamonds adsorb aflatoxin B_1_ from aqueous solutions at different pH, were confirmed by in vivo experiments with rats [83]. In order to confirm the effectivity and safety of this adsorbent on animal species, further studies including well-designed in vitro trials are needed. The practical and economic feasibility aspects should also be taken into account [82].

The formation of bonds between polymers, such as cholestyramine, divinylbenzene-styrene and polyvinylpyrrolidone, and mycotoxins were confirmed in vitro and in vivo [52]. Cholestyramine is a binding resin that has proven to bind bile acids in the gastrointestinal tract and decrease low-density lipoproteins and cholesterol. It has been shown that cholestyramine is an efficient binder for ochratoxin A, fumonisins and zearalenone in vitro [52,57,61]. Efficacy of cholestyramine in the dietary concentration of 2% on top of compound feed was confirmed in experiments with feed contaminated by zearalenone and by the biomarker assay in vivo in laboratory animals for fumonisins [52,57,61]. Cholestyramine efficacy for detoxification of zearalenone was also confirmed during other studies on laboratory animals [84]. A polyvinylpyrrolidone, a synthetic water-soluble polymer, was researched as a binder for mycotoxins as well [85]. It was shown that polyvinylpyrrolidone is able to bind aflatoxin B_1_ and zearalenone, while it did not alleviate the toxicity of deoxynivalenol in pigs. It should be noted that the high costs of polymers are a limiting factor for its practical applications.

The mycotoxin-sequestering capacity of different high-fiber feedstuffs, such as hays (e.g., alfalfa hay) or straws (e.g., wheat straw), was recognized a long time ago, but there are principally practical experiences, e.g., in equine nutrition, without scientific assessment. The positive effect of alfalfa fiber was first proved against zearalenone in laboratory animals and pigs and also against T-2 toxicosis in laboratory animals, respectively [86,87,88,89]. However, it should also be mentioned that, besides its positive effects, alfalfa fiber is a potential source of *Fusarium* contamination, and its high inclusion rates (15–25%) required in the diet may cause digestive-physiological disturbances. Micronized wheat fiber has recently been found effective in decreasing the accumulation of ochratoxin A in laboratory animals’ liver and kidney tissues. When used at an inclusion level of 20 kg/t, it significantly increased the excretion of ochratoxin A via the feces [90,91]. Recently, Avantaggiato et al. [92] showed that a red-grape pomace (pulp and skin) can sequester distinct mycotoxins quickly and simultaneously. Aflatoxin B_1_, followed by zearalenone, ochratoxin A and fumonisin B_1_, was the most affected mycotoxin. In pigs, using a urinary biomarker method the effectiveness of grape pomace in secreting mycotoxins has been confirmed [79]. Aflatoxin B_1_ (67%) and zearalenone (69%) considerably lowered the urinary mycotoxin biomarker of the grape pomace. Taking these outcomes into consideration, the authors indicated that the use of grape pomace as a large-spectrum adsorbent material has its potential. Greco et al. [93] recorded evidence on the capacity of food plants and by-products other than grape pomace and wheat fibers to absorb mycotoxins. The research results are highly innovative and prove that a wide range of mycotoxins is also available in some dietary fibers. Aflatoxin B_1_, zearalenone, and ochratoxin A were most of the adsorbed mycotoxins. Adsorption of aflatoxin B_1_, zearalenone, and ochratoxin A was not impacted by pH, and the adsorbed fraction was not released when acid-to-neutral pH increased. Mycotoxin fumonisin B_1_ has been adsorbed to a lesser level in this research and its adsorption has been affected by a medium’s pH.

Polymeric humic materials comprise several binding sites and are being incorporated into humans as a compound to minimize bacterial endotoxins absorption and systemic accessibility. A high-quality humic acid derivative, called oxyhumate, has been reported to have the mycotoxin-sequestering capacity and recommended for use against aflatoxicosis based on in vivo studies in chickens [94]. The excellent connection capability of humic substances with zearalenone was an exciting finding, as assessed in vitro research [60]. These compounds should, therefore, be further tested in vivo.

The other groups of fiber components are the cell wall components of the yeast *Saccharomyces cerevisiae*, the mannan-oligosaccharides (MOS) or their esterified form with β-D-glucan (esterified glucomannan), which showed the considerable binding ability for several mycotoxins in vivo. *Saccharomyces cerevisiae* and lactic acid bacteria are the two most important food fermentation microorganisms that have proven to bind various mycotoxins [20,54,95]. The reversible and strain and dose-dependent phenomenon of mycotoxin binding of some chosen lactic acid bacteria was outlined and did not influence the viability of the lactic acid bacteria. It should be noted that there may be a relationship between lactic acid bacteria and the accumulation of mycotoxins through two particular procedures like binding and biosynthesis inhibition. There could, therefore, be a high value for the reduction of mycotoxin exposure for lactic acid cultures with a strong anti-fungal, anti-mycotoxigenic and mycotoxin potential [96].

Fungal conidia can bind mycotoxin individually or together (between 29 and 60%), particularly zearalenone and ochratoxin A [97]. *Saccharomyces cerevisiae* live yeast was shown to reduce the detrimental effects of aflatoxin in broiler diets [20,34,95,98,99]. The aflatoxin protective effect of live yeast was confirmed in rats, but thermolyzed yeast was shown ineffective. The potential to bind several mycotoxins was shown as fibrous material from the cell wall of the yeast. It has been shown that esterified glucomannan polymer obtained from the yeast cell wall separately and in combination binds aflatoxin, ochratoxin and toxin T-2. Additions of 0.5 or 1.0 kg/t doses of esterified glucomannan to aflatoxin-contaminated diets resulted in broiler chicks, with dose-dependent reactions. Similarly, in relation to aflatoxin-contaminated diets of dairy cows, esterified glucan polymer considerably decreased residues of aflatoxin in milk. The esterified glucan polymer may have the capability to bind several mycotoxins. A glucan polymer-bound both T-2 toxin and zearalenone in vitro, and it was protective against depression in antioxidant activities resulting from T-2 toxin consumed by growing quail. A glucan polymer product has protected swine, broilers, and hens against some of the detrimental effects of multiple mycotoxins, while another glucan polymer product did not alleviate the toxic effects on mink consuming diets contaminated with fumonisin B_1_, ochratoxin A, moniliformin and zearalenone [98,99]. These polysaccharides, in addition to binding mycotoxins, also provide other functions to regulate the damage of mycotoxins in animal organs, including modulation of immune operations and binding gastrointestinal pathogens [100,101,102]. It should also be noticed that many trials are carried out using commercial products which may not consist exclusively of glucomannans, but contain tiny quantities of aluminosilicates that are specifically added to bind aflatoxins.

Numerous studies and several comprehensive reviews as above demonstrate the increasing interest in the use of mycotoxin-detoxifying agents as technological feed additives.

The best way to evaluate mycotoxin binders is with in vivo experiments. Naturally, in vivo models are perfect and hard to conduct in theory. It is complicated, costly, and time-consuming to collect the definitive data. Individual bioassays with the same strain, age, body weight, and dietary type should take place in vivo research to achieve coherent outcomes. Differences in farm conditions and types, health, development, and maturity of animals may also have an effect on outcomes. Binders with varying rates of incorporation, distinct mycotoxins, animal species, age, gender, and environment should be assessed as well. Moreover, according to the EU Guideline 2001/79/EC on additives for use in animal nutrition [50], the in vivo efficacy of binders should be proven by using an experimental design justified according to the claim for the use of the additive, and by using specific biological markers such as tissue residues or changes in biochemical parameters [103]. The chosen biomarker for exposure should be specific for each mycotoxin and target species, closely related to exposure and easy to detect with sensitive analytical methods validated for the matrix used [3]. EFSA has proposed different biomarkers for exposure to aflatoxin B_1_, deoxynivalenol, zearalenone, ochratoxin A, and fumonisins. To date, the majority of the research on the effectiveness of mycotoxin binders has only been done on the feed consumption and measurements of performance. Few in vivo research papers have explored potential side impacts on animal performance or the health of these agents.

## 6. Biological Detoxification and Biotransformation

The application of microorganisms or enzyme systems to contaminated feeds can detoxify mycotoxins by metabolism or degradation in their gastrointestinal tract. This process is an irreversible and environmentally friendly method of detoxification, as it does not leave toxic residues or unwanted by-products. A large number of detoxifying studies were performed in the 1980s and 1990s on ochratoxin A, trichothecenes and zearalenone [32,46,98,104,105,106]. While initial in vitro reports of the microbial mycotoxins detoxification can be dated back to the 1960s, until now, only a few organically transforming agents mainly microorganisms were tested in vivo for their efficacy.

Aflatoxin degradation in laboratory conditions has been investigated in numerous cases over the years [107,108,109,110], but there is currently no biological system for the entire commercial sphere to be used. Interesting results have been obtained for *Nocardia corynebacteroides* application. This soil bacterium is supposed to remove aflatoxins B, G, and M_1_ from a variety of food products, including milk, oil, peanut butter, peanuts, and maize, without leaving any toxic by-products. It has been shown to be effective in the irreversible removal of aflatoxin B_1_ from aflatoxin-contaminated compound feed for broiler chicken nutrition [111].

In the last 20 years, many tests on trichothecenes with cows’ rumen, gastrointestinal experimental or soil in the laboratory were conducted in vitro. Microbial biodegradation of trichothecenes via various pathways such as oxygenation, de-epoxidation, epimerization, and glucosylation has been elucidated [106]. Rumen fluid is selected due to knowledge of the fact that ruminants are very resistant to the toxic effects of mycotoxins, such as deoxynivalenol and trichothecenes. Deoxynivalenol’s 12,13-epoxy-ring and T-2 toxin seem to be a part of the toxicity molecule. The mycotoxins are less toxic when opening up this ring. The *Eubacterium* sp. strain BBSH 797 has been developed into a commercial product for detoxifying trichothecenes in animal feed [98,105]. There are few studies on the transformation of toxins of T-2 and HT-2. Certain metabolites had been de-acetylated and de-epoxidized T-2. Some microorganisms could transform T-2 into HT-2, but detoxification has not been recorded.

Some bacteria, molds, yeasts, and plants can transform ochratoxin A into a less toxic compound which has been confirmed in many scientific reports [51,112,113]. These changes lead to phenylalanine formation [32,46,98,104,105,106,114]. *Aspergillus*, *Rhyzopus* and *Penicillium* spp. are particularly effective for ochratoxin A removal [113]. As a biologic controller in wine, *Aureobasidium pullulans* prevents the accumulation of ochratoxin A in the grape and decreases the symptoms of *Aspergillosis*. Plants, such as wheat and maize, or fungi, such as *P. ostreatus*, are capable of removing ochratoxin A but no transformation products have been identified [115]. Moreover, a new yeast strain that can degrade ochratoxin A and zearalenone was isolated and characterized. This strain was called *Trichosporon mycotoxinivorants* because of its property for degrading these mycotoxins. A feeding trial, which tested the efficacy of *T. mycotoxinivorans* to suppress ochratoxicosis, proved that the dietary inclusion of this yeast blocks ochratoxin A induced immune suppression in broiler chicks. *T. mycotoxinivorans* have been recognized as the principal transformation product of zearalenone. The structure of the metabolite, ZOM-1 is characterized by the opening in the group of ketones in C6 of the macrocyclic ring of zearalenone [116]. Even at concentrations 1000-fold higher that zearalenone, the ZOM-1 did not show estrogen activity in a sensitive yeast bioassay and did not interact with the human estrogen receptor in the competitive in vitro binding experiment [116].

Fumonisin B_1′_s main amine is responsible for its toxicity. Thus, this molecule’s deamination would significantly decrease its toxicity. Very few biodegradation studies for fumonisin B_1_ have been conducted. The main microorganism capable of degrading fumonisin B_1_ is the black yeast, *Exophiala spinifera*. The transformation of fumonisin B_1_ into amino polyol AP_1_ is performed by an extracellular carboxylesterase. This enzyme has been cloned and proven effective in transgenic maize since the plant became fumonisin resistant [117]. Two genes that cause fumonisin B_1_ degradation by the bacterium *Sphingopyxis* sp. in the latest study have been recognized, isolated and expressed in heterological terms. The researchers found that the successive effect of these gene-encoded enzymes to detoxify fumonisin B_1_ [118]. It is important to mention that molecular oxygen is not necessary for the operation of the mentioned enzymes. The results of this study, therefore, provide the foundation for the development of an enzyme detoxification process for fumonisin B_1_ in animal feed.

Although numerous papers on biological transformation by microorganisms of mycotoxins are present, they were limited in their use for feed detoxifying. This may result from a lack of understanding about the conversion process, the toxicity of the products being transformed, the impact of conversion responses on nutritional values for feed and animal safety. Biological agents used as feed additives should generally degrade mycotoxins into non-toxic metabolites under various environmental conditions. They must be safe and stable in the gastrointestinal tract of animals. To date, few micro-organisms meet these needs.

Dietary manipulations include improved dietary ingredients, dietary supplementation or additives with toxicity-protective characteristics or the addition of no-nutrient sequestrants to reduce mycotoxin bioavailability, in order to decrease mycotoxin induced intake [20,44,52].

The control of multiple metabolic route ways has an influence on protection from stress, which may be attributed to the sensitive equilibrium between antioxidants and pro-oxidants. Diet antioxidants like vitamin E, carotenoids and selenium can regulate this equilibrium [119,120,121,122]. On the other side, this antioxidant/prooxidant equilibrium has an adverse effect on dietary stress variables. In this regard, mycotoxins are regarded as one of the main stress variables in feed [121,123,124,125]. In fact, enhanced supplementation for antioxidants can safeguard against mycotoxins’ poisonous behavior. The antioxidant characteristics of selenium and retinol, ascorbic acid, tocopherol, and their precursors are known to operate as free radical scavengers and to safeguard mycotoxin from membrane harm [126]. Certain antioxidants, selenium, and vitamins can also induce or boost liver and other tissue detoxication mechanisms, thereby increasing mycotoxin detoxification. Food elements in coffee, strawberries, tea, pepper, raisins, turmeric, tonka beans, garlic, chocolate, and onions achieved attractive outcomes. Furthermore, certain medicinal herbs and plant extracts could possibly protect them from aflatoxin B_1_, fumonisin B_1_, and ochratoxin A [121,127,128,129]. However, there is much less information on other mycotoxins, primarily from in-vitro research and focusing on aflatoxin B_1_. For the assessment of their practical benefits further feeding studies with antioxidants and vitamins with farm animals are necessary. The choice of the most appropriate nutritional methods requires knowledge of the type of antioxidants in the diet, their bioavailability and food sources, and the exact intake required to achieve these protective effects. In addition, a mixture of natural antioxidants (e.g., medicinal plant extracts, essential oils, herbs, spices, some vitamins, etc.) with feed additives that act as detoxifiers of mycotoxins could be a further step in the fight against mycotoxicosis in animal production.

A similar tendency in research was noticed with regards to the usage of ozone in laboratory test animals for the prevention of zearalenone estrogenic effects [130]. Trichothecenes’ biological activities were also altered by oxidation, with ozone most probably attacking trichothecenes‘ double bond. Recently, essential oils, natural-based or natural identical, have been tested for their efficacy to reduce ochratoxin A contamination in feed [8,131,132]. Ochratoxin A is found in various kinds of feed such as cereals, coffee, beans and foods such as dried fruits, grapes, wines, and their derivatives [39]. Nephrotoxic, cancer-genic, immunotoxic, teratogenic and genotoxic activities become noticeable when animals ingest contaminated feed [112]. In Puvača et al.’s [114] in vitro research, the influence of tea tree essential oil (*Melaleuca alternifolia*) on ochratoxin A fungal synthesis has been verified. The essential oil’s (7.5; 15.0 and 30.0 μg/mL) decontamination potential was assessed on the growth of ochratoxin A producer (*Aspergillus niger*). The production of ochratoxin A in the presence of the essential oil depended on the incubation temperature, 20 and 30 °C. The values obtained from 20 °C showed a reduction in ochratoxin A synthesis by A. niger ranging from 53.87% to 96.22% while 18.36% to 72.85% at 30 °C. Based on the obtained results Puvača et al. [114] concluded that essential tea tree oil could serve as a prospective biocontrol agent for contamination of ochratoxin A in feed and compound feed.

## 7. Conclusions

In conclusion, levels of particular mycotoxins in feeds have been reduced, but, so far, no single technique has been established that is equally efficient against the broad variety of mycotoxins that can co-occur in various commodities. Furthermore, procedures of detoxication that appear to be efficient in vitro will not necessarily maintain their effectiveness in an in vivo test. Therefore, further research on the stability of toxins in the whole “field to fork” chain is required in order to have general recommendations for the reduction of these adverse contaminants and to avoid re-contamination. Research on mycotoxins in feeds and their potential interactions should be carried out simultaneously, and the solutions concerning how the toxicological importance of such interactions could be evaluated and practically used. There is increasing business interest in the use of feed additives to avoid mycotoxin absorption and the toxic impacts on farm animals. There are also new products on the market available and some of them have been in wide use for several years. The efficacy of the additives for the distinct mycotoxins and livestock must be proved, e.g., by means of peer-checked research. It is recommended that cell lines or artificial models be used in the simulation instead of living experimental animals, questioning the animal’s welfare.
